# Advancing Public Health Infrastructure for Dementia Through the Public Health Centers of Excellence

**DOI:** 10.1093/geroni/igaf122.4273

**Published:** 2025-12-31

**Authors:** Shelby Roberts, Joseph Gaugler, Joshua Chodosh, Soo Borson, Mickal Lewis

**Affiliations:** Alzheimer’s Association, Chicago, Illinois, United States; University of Minnesota, Minneapolis, Minnesota, United States; NYU Langone, New York City, New York, United States; University of Southern California, Los Angeles, California, United States; Alzheimers Association, Chicago, Illinois, United States

## Abstract

Over the past five years, the three Public Health Centers of Excellence (PHCOEs) have advanced the national public health response to Alzheimer’s and related dementias by translating research into practice, building capacity, fostering innovation across diverse jurisdictions, and providing critical support to both BOLD program awardees and health departments operating without federal funding. The PHCOE on Dementia Risk Reduction, housed at the Alzheimer’s Association, has synthesized scientific evidence on modifiable risk factors for dementia into short research briefs for health department officials and trained 64 local health department officials in 38 states and the District of Columbia. The PHCOE on Early Detection, housed at New York University’s Grossman School of Medicine, has addressed the systemic underdetection of dementia, collaborating with over 75 organizations. It has developed toolkits and resources to promote early and equitable detection, delivered 61 presentations, and catalyzed national dialogue on making early detection routine practice. The PHCOE on Dementia Caregiving, housed at the University of Minnesota, has increased awareness of caregiving as a public health priority through the dissemination of toolkits, hosting webinars and virtual panels, and participation in 37 national conferences. Collectively, the Centers have reached over 14,000 professionals, hosted three national conferences, and laid the groundwork for sustained public health action on dementia. These efforts have catalyzed the integration of dementia-related strategies into public health practice, fostering innovation, collaboration, and evidence-based action.

